# Editorial: Gastrointestinal and bilio-pancreatic stenting: when, which stent and why

**DOI:** 10.3389/fgstr.2023.1271139

**Published:** 2023-08-21

**Authors:** Andrea Tringali, Theodor Voiosu, Michael Fernandez Y. Viesca, Christian Gerges

**Affiliations:** ^1^ Digestive Endoscopy Unit, Fondazione Policlinico Gemelli IRCCS, Università Cattolica del Sacro Cuore, Roma, Italy; ^2^ Department of Gastroenterology, Colentina Clinical Hospital, Bucharest, Romania; ^3^ Hepato-Pancreatology and Digestive Oncology Department, Hôpital Erasme, Université libre de Bruxelles, Brussels, Belgium; ^4^ Evangelisches Krankenhaus (EVK) Duesseldorf, Duesseldorf, Germany

**Keywords:** plastic stent (PS), SEMS (self-expandable metallic stent), gastrointestinal stenting, bilio-pancreatic stenting, LAMS- lumen-apposing metal stents

Currently, endoscopic stenting is a standardized procedure to treat benign and malignant strictures of the gastrointestinal tract including the bilio-pancreatic ductal system.

The goal of this Research Topic is to provide a practical summary regarding indications and techniques of endoscopic stenting.

The Research Topic included two review articles (Conrad and Ellrichmann; Cappello et al.) and two research articles (Zhang et al.; Bi et al.) underlying that the field of endoscopic stenting is still expanding with innovative devices.

Conrad and Ellrichmann described indications to pancreatic stenting providing a detailed overview on different use of pancreatic stenting in the setting of therapeutic and prophylactic applications; different stent shape and size are available and the choice should be tailored case by case. Pancreatic stents are commonly plastic, while Self Expandable Metal Stents (SEMS) are still under investigation to drain pancreatic duct strictures in the setting of chronic pancreatitis due the high rate of complications described (migration, “*de novo*” strictures). New biodegradable stents have promising results for pancreatic endotherapy and further data are needed to propose their application in clinical practice. Lumen Apposing Metal Stents (LAMS) were introduced 10-years ago and represent a milestone in the drainage of pancreatic fluid collections complicating acute pancreatitis. LAMS are placed safely under EUS-control expanding the indications to the treatment of post-pancreatitis complications.

The authors provided also a description of stents characteristics and deployment technique.

Cappello et al. had a complete overview concerning trans-papillary biliary stenting performed during ERCP. Biliary strictures are more frequently drained with SEMS especially if malignant; efficacy of SEMS is superior to plastic stents. In the setting of benign biliary strictures multiple plastic stents are still a first line option especially to treat post-operative complications (liver transplantation and cholecystectomy), but fully covered SEMS are expanding and are becoming a fist line option like in the setting of biliary strictures secondary to chronic pancreatitis. Fully covered SEMS are also essential to treat ERCP-related complications like retroperitoneal perforations and massive post-sphincterotomy bleeding. Indications to biliary stent are summarized in figures where current guidelines are presented in “graphical abstracts”.

The other articles describe innovative applications of colonic (Zhang et al.) and esophageal (Bi et al.) stents. Zhang et al. evaluated colonic stents to treat acute left-sided malignant colonic obstructions as a bridge to surgery: interestingly a delayed surgical resection (> 4 weeks) resulted in fewer complications and shorter postoperative recovery.

Bi et al. report a series of 17 patients where esophageal stents were successfully used to obtain compression hemostasis in bleeding esophageal cancers; this is an interesting salvage therapy in selected cases. This paper represents the evolution of stenting: initially esophageal stents had the unique indication to bypass a stenosis allowing oral feeding; changes in stents design and covering characteristics lead to the treatment of refractory gastrointestinal bleeding, as reported.

Gastrointestinal and bilio-pancreatic stenting had an impressive development during the last two decades. Today the choice of endoscopic stents can be difficult, due to the wide variations of devices available. The aim of the present Research Topic is to provide to the clinician a practical approach to endoscopic stenting.

## Author contributions

AT: Writing – original draft. TV: Writing – review & editing. MV: Writing – review & editing. CG: Writing – review & editing.

